# Compost Grown *Agaricus bisporus* Lacks the Ability to Degrade and Consume Highly Substituted Xylan Fragments

**DOI:** 10.1371/journal.pone.0134169

**Published:** 2015-08-03

**Authors:** Edita Jurak, Aleksandrina Patyshakuliyeva, Ronald P. de Vries, Harry Gruppen, Mirjam A. Kabel

**Affiliations:** 1 Wageningen University, Laboratory of Food Chemistry, Bornse Weilanden 9, 6708 WG, Wageningen, The Netherlands; 2 Fungal Physiology, CBS-KNAW Fungal Biodiversity Centre & Fungal Molecular Physiology, Utrecht University, Uppsalalaan 8, 3584 CT Utrecht, The Netherlands; Iowa State University, UNITED STATES

## Abstract

The fungus *Agaricus bisporus* is commercially grown for the production of edible mushrooms. This cultivation occurs on compost, but not all of this substrate is consumed by the fungus. To determine why certain fractions remain unused, carbohydrate degrading enzymes, water-extracted from mushroom-grown compost at different stages of mycelium growth and fruiting body formation, were analyzed for their ability to degrade a range of polysaccharides. Mainly endo-xylanase, endo-glucanase, β-xylosidase and β-glucanase activities were determined in the compost extracts obtained during mushroom growth. Interestingly, arabinofuranosidase activity able to remove arabinosyl residues from doubly substituted xylose residues and α-glucuronidase activity were not detected in the compost enzyme extracts. This correlates with the observed accumulation of arabinosyl and glucuronic acid substituents on the xylan backbone in the compost towards the end of the cultivation. Hence, it was concluded that compost grown *A*. *bisporus* lacks the ability to degrade and consume highly substituted xylan fragments.

## Introduction

Commercially, white button mushrooms (*Agaricus bisporus*) are grown on compost, of which the carbon and nitrogen sources may differ throughout the world. In Europe, *A*. *bisporus* compost is based on wheat straw, and horse and chicken manure. Before mushroom mycelium is introduced two composting stages, having different conditions with respect to e.g. pH and temperature, are applied to make the compost accessible and highly specific for the growth of *A*. *bisporus* [1,2]. Mycelium is introduced to the compost and grown until the compost is considered mature. This mature compost contains about 27% (w/w) of carbohydrates based on total dry matter [1]. A casing layer (mixture of peat and lime) is put on top of the mature compost to induce fruiting body formation [3]. Normally, several flushes of mushrooms can be harvested before the compost is considered spent. This spent compost still contains about 10% (w/w) of carbohydrates based on total dry matter [2], which include both plant and fungal biomass carbohydrates. Apparently, complete degradation and consumption of compost carbohydrates is not achieved, but why this fractions remains has so far not been investigated in detail.

In a previous study, it was shown that before mushroom mycelium is introduced to the compost, mainly lowly substituted xylan and cellulose are present as carbohydrates. This xylan (34 mol%) is substituted with arabinosyl (5 mol%) and with 4-*O*-methyl-glucuronic acid (4 mol%) residues, while hardly any ester bound substituents are present [1]. Already during mycelium growth of *A*. *bisporus* these xylan and cellulose are partly degraded and consumed [2]. It is likely that such modifications of the available carbohydrate structures affect their degradation during the later fruiting body formation stages.

**Fig 1:**
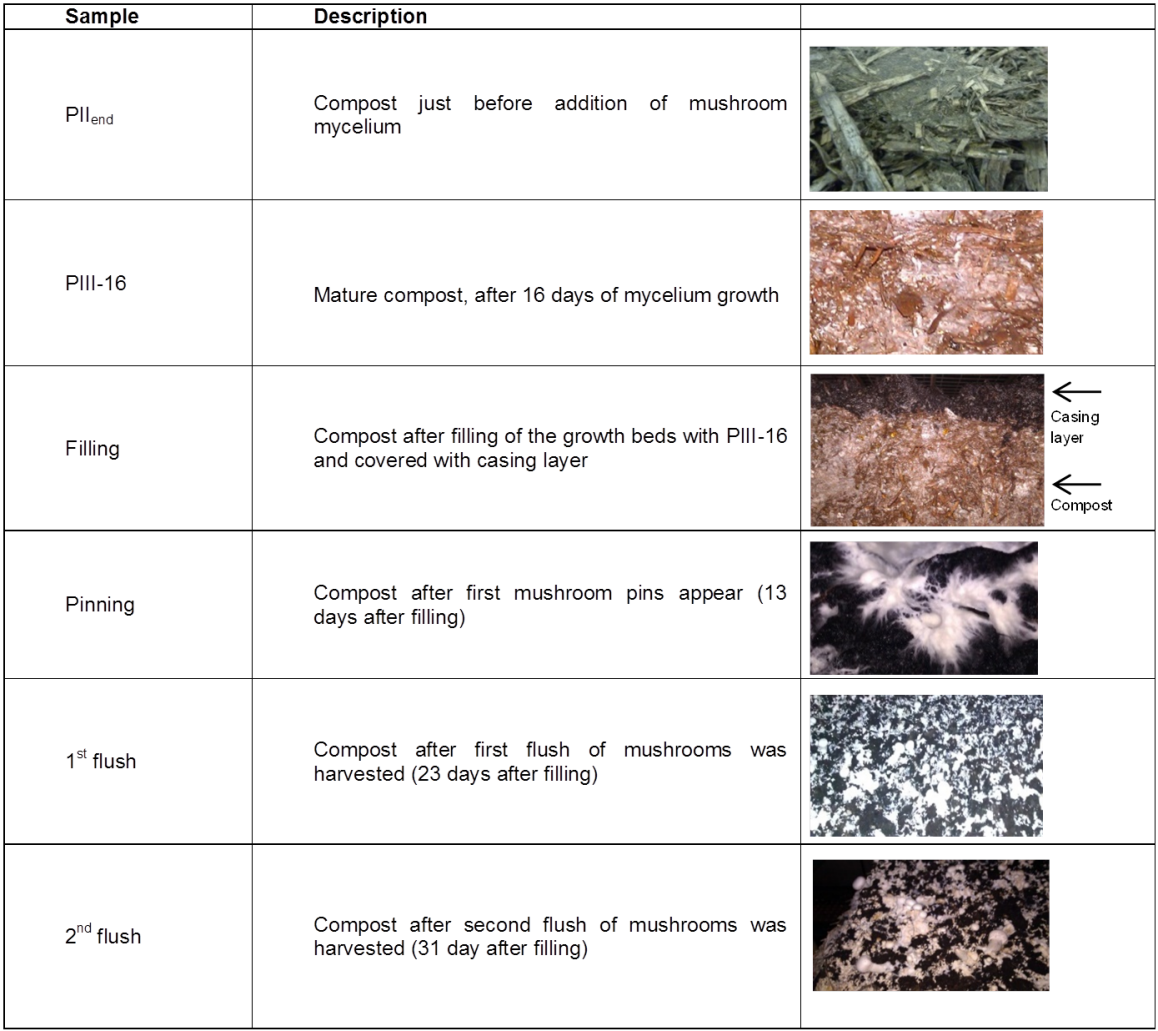
Compost sample codes and description.

The changes in carbohydrate structure and content are expected to be majorly related to enzyme activities produced by *A*. *bisporus*, since mature compost is fully colonized with mycelium of this species. Others have already studied enzyme activities in compost during mycelium growth and fruiting body formation [4–6]. However, the main focus of these studies was on lignin degrading enzymes or on exo-acting carbohydrases. In addition, these exo-acting enzymes were investigated at their optimal conditions with standard assays, which are not always representative for their activity under composting conditions and on compost carbohydrates [7]. In this paper we present the first thorough characterization of enzyme activities present in compost during mycelium growth and fruiting body formation of *A*. *bisporus* at compost conditions, which would not necessarily be considered optimal for the best activity of these enzymes. A deeper analysis was facilitated by the availability of the *A*. *bisporus* genome sequence [8] and a recent study in which all genes encoding (putative) carbohydrate degrading enzymes have been identified [9]. In the same study it was described which of these genes are up- or down- regulated during fruiting body formation. Nevertheless, it is not known whether these expressed genes are translated and whether their corresponding enzymes are active in compost. Therefore, in the study presented here the aim is to investigate which enzymatic activities are present during the growth of *A*.*bisporus* in compost at different time points and compare these both to the genome information described by Patyshakuliyeva et al. (2013) [9] and to the structure of the remaining carbohydrates in the compost. This provides a deeper understanding of the carbohydrate degrading machinery of *A*. *bisporus* and the commercial cultivation process of this fungus. Moreover, this knowledge is expected to provide possible ways for improving the carbohydrate utilization by *A*. *bisporus* and consequently mushroom production. The samples used in this study correspond to different stages of the cultivation (Fig 1).

## Materials and Methods

### Compost samples

The strain used in this study is the commercial *A*. *bisporus* strain Sylvan A15, (Sylvan, Kittanning, PA, USA). For a first screening of enzyme activities, compost samples of PIIend and PIII-16 were supplied by CNC as described previously [1]. For a second, more extensive screening, fresh compost samples of end of Phase II (PIIend), which is the compost phase just before addition of mycelium and of 16 days mature mycelium grown compost (PIII-16) were obtained. Further, composts obtained after filling of the beds with PIII-16 compost and covered with casing soil (Filling), after pinning of *A*. *bisporus* (Pinning), after the first flush (1^st^ flush) and after the second flush (2^nd^ flush) of mushrooms were supplied by CNC; all were from the same timeline. Sample codes and description are summarized in Fig 1. Compost samples (about 1 kg each) were collected from the same initial preparation (same initial bach) in duplicate, taken from the top layer (layer of 10 cm) excluding the casing layer and frozen. For carbohydrate analysis, part of the frozen samples were freeze dried and milled (<1 mm) (Mill MM 2000, Retsch, Haan, Germany). After milling, duplicates were mixed in equal ratios and the mixed samples were analyzed.

### Extraction of enzymes

Extraction of enzymes from the compost was tested under various conditions such as pH, extraction time and temperature and, finally, a modified method from Singh et al. (2003) [10] was used in order to achieve the highest recovery of proteins from the compost.

Frozen compost samples were defrosted and on the same day, 10 g of the sample was mixed with 100 mL distilled water in 250 mL Erlenmeyer flasks. The flasks were incubated for 1 h at 200 rpm and 4°C. Samples were then centrifuged (10 000 x g, 15 min, 4°C) and the supernatant was collected as the crude enzyme extract, which was used for PNP assays (2.4). For the other assays, supernatants were filtered through 0.2 μm filters. The filtrate obtained was then filtered through a 10 kDa filter (Merck Millipore, Billerica, MA, USA) and washed twice to remove small carbohydrates. The 10 kDa retentate was mixed with millipore water to reach the starting volume and denoted as “enzyme extract" from corresponding compost phases.

### Protein content, pH, conductivity and Mw profiles

The protein content of the prepared enzyme extract was measured by using the bicinchoninic acid (BCA) assay with bovine serum albumin as standard (Pierce, Thermo Scientific, Rockford, IL, USA).

Extracellular proteins in the enzyme extracts were separated by SDS-PAGE using 12% polyacrylamide gels and stained using GelCode Blue stain reagent (Pierce, Rockford, IL, USA).The enzyme extracts were concentrated 4 times and 40 **μ**L of concentrated extract was loaded on the gel.

Conductivity was measured by the conductivity meter (WTW inoLab pH/Cond 720, Weilheim, Germany) and pH by pH meter (WTW inoLab 7110, Weilheim, Germany).

### Enzyme activity assays

Exo-enzyme activities in the enzyme extracts were measured in duplicate using p-nitrophenol (PNP)-linked substrates (4-nitrophenyl α-L-arabinofuranoside, 4-nitrophenyl β-D-glucopyranoside, 4-nitrophenyl β-D-xylopyranoside, 4-nitrophenyl β-D-cellobioside, and 4-nitrophenyl β-D-mannopyranoside; Sigma-Aldrich, St. Louis, MO, USA). In a total volume of 100 μL using 40 μL of the compost extract, 10 μL of 0.01% (w/v) p-nitrophenol-linked substrates, and 50mM sodium acetate buffer (pH 5.0) was mixed. Samples were incubated in microtiter plates for 4 h at 30°C. Reactions were stopped by addition of 100 μL 0.25M Na_2_CO_3_ solution. Absorbance was measured at 405 nm in a microtiter plate reader (FLUOstar Optima; BMG LabTech, Ortenberg, Germany). The activities were calculated using a standard curve ranging from 0 to 20 nmol of p-nitrophenol per assay volume. Overall, exo-enzyme activities of compost enzyme extracts were observed to be quite low (between 0.1 and 4.5 nmol pnp ml^-1^ min^-1^) after 4 h.

Enzyme activities were tested for their overall hydrolytic activity (combined exo- and endo-activity) on various polysaccharides, and assayed by the PAHBAH reducing-end assay in duplicate [11]. For this assay, a working solution was prepared by mixing one part of p-hydroxybenzoic acid hydrazide (5% w/w) in 0.5M HCl with four parts of 0.5M NaOH. The sample (10 **μ**L) was mixed with 200 **μ**L working solution and incubated at 70°C for 30 min in microtiter plate covered with aluminum foil. After cooling, absorbance was measured at 405 nm. The reducing-end concentration was quantified using xylose and glucose calibration curves (10–750 **μ**g ml^-1^).

Endo-enzyme activities were tested on a range of carbohydrate substrates. Wheat arabinoxylan (medium viscosity), birchwood xylan and potato galactan were obtained from Megazyme (Wicklow, Ireland). Tamarind xyloglucan was obtained from Danippon Pharmaceutical (Osaka, Japan) and carboxymethyl cellulose (low viscosity) was obtained from Sigma-Aldrich (St. Louis, MO, USA). Rhamnogalacturonan I (apple modified hairy regions-B, saponified) were obtained as described by Schols et al. (1990) [12]; Branched sugar-beet arabinan was obtained from British Sugar (Peterborough, UK [13]). Finally, low (DM 30, C30) and high (DM 70, C72) methylated homogalacturonan was provided by Copenhagen Pectin A/S (Lille Skendved, Denmark, [14]). These carbohydrate substrates were incubated with enzyme extracts from PIIend and PIII-16 in water. For incubation, 800 **μ**L of 2.5 mg ml^-1^ of substrates and 200 **μ**L of non-diluted enzyme extract was used. Incubations were performed at 35°C rotating “head over tail” for 24 h.

Substrates for the second screening were Locus bean gum (SKW Biosystems, Enschede, The Netherlands), wheat arabinoxylan (WAX, Medium viscosity, Megazyme), birchwood xylan (Megazyme), carboxymethylcellulose (Low viscosity, Sigma-Aldrich) and a well-defined digest of WAX by endo-xylanase 1 from *A*. *awamori* [15,16]. Substrates were incubated in water with enzyme extracts from PIIend, PIII-16, Filling, Pinning, 1^st^ flush and 2^nd^ flush. Incubations were performed as described above.

In order to confirm the presence of double substituted xylo-oligomers in the WAX digested with compost-extracts, WAX was first incubated with 1^st^ flush, and sequentially incubated with pure GH43 AXH-d3 arabinofuranosidase [17] as described above.

### Analytical methods

*Neutral carbohydrate and uronic acid content and composition* were determined in duplicate, as described in [1].

*High Performance Size Exclusion Chromatography (HPSEC)* was performed as described in [1]. Enzyme digests were analyzed without prior dilution. Enzyme activity was evaluated by comparing the high performance size exclusion chromatography (HPSEC) elution profiles of polysaccharides before and after enzyme hydrolysis. If after incubation with the enzyme extracts the HPSEC profile was the same as in the substrate without enzymes it was concluded that there was “no degradation”. When a decrease in large molecular weight (Mw) material was observed together with the formation of some smaller Mw weight material after enzyme hydrolysis it was annotated as “partial degradation”. When none of the originally high Mw material of the polysaccharide tested remained, but only small Mw material was observed after incubation with enzyme extracts, it was annotated as “complete degradation” (Table 1).

**Table 1:** First screening of extracellular enzyme activities from the compost in PIIend and PIII-16 analyzed by HPSEC after 24 h incubation.

Substrate	PII_end_	PIII-16
Low methylated homo-galacturonan (DM30)	-	-
High methylated homo-galacturonan (DM70)	-	-
Sugar beet branched arabinan	-	+
Rhamnogalacturonan I (RGI)	-	-
Potato galactan	+	+
Wheat arabinoxylan (WAX)	++	+
Galactomannan	+	+
Carboxymethyl cellulose (CMC)	+	-
Xyloglucan	+	+

(- no degradation, + partial degradation, ++ complete degradation)

*High Performance Anion Exchange Chromatography (HPAEC)* was performed as described in [1]. Oligosaccharides released from WAX were identified using a profile of WAX degraded by pure, well characterized endo-xylanase was used [18]. Enzyme digests were diluted 10 times prior to analysis.

## Results and Discussion

### Preliminary screening of enzymes from extracts of PIIend and PIII-16

A first screening was performed for two enzyme extracts, PIIend and PIII-16, on nine different cell wall polysaccharides. Degradation of these polysaccharides was evaluated by comparing the high performance size exclusion chromatography (HPSEC) elution profiles of polysaccharides before and after enzyme hydrolysis. The results are summarized in Table 1. In PIIend detected enzymatic activities are expected to originate from the microbiota present in the compost and in PIII-16 apart from the microbiota *A*. *bisporus* mycelium is present and a change in enzymatic activities is likely dominated by *A*. *bisporus*.

Wheat arabinoxylan (WAX) was completely degraded by extracts from PIIend and partially by PIII-16. This was expected as xylan is, next to cellulose, the main carbohydrate source in compost used for mushroom growth [1]. In addition, cellulose (CMC) was partially degraded by PIIend-extract, whereas not at all by PIII-16-extract. For the pectic substrates, almost no degradation was observed, apart from branched arabinan by PIII-16-extract. Further, partial degradation was observed by both extracts from PIIend and PIII-16 on model galactomannan and xyloglucan. Recently it was shown that the *A*. *bisporus* genome encodes enzymes targeting all plant polysaccharides [9], likely due to the fact that in nature *A*. *bisporus* grows on a variety of (plant-based) substrates. This correlates with the observed enzyme activities towards xylan, arabinan, galactan, mannan and xyloglucan in PIII-16 (Table 1). However, no activity towards homogalacturonan, rhamnogalacturonan I (RGI) or CMC was observed. Previously, genes encoding pectin and cellulose degrading enzymes were found to be upregulated in the compost at the stage of the first flush of *A*. *bisporus* [9] it remains unclear whether these pectin degrading enzymes are produced. It is likely that production of specific polysaccharide degrading enzymes in *A*. *bisporus* respond to the presence of different polysaccharide-derived inducers in the growth medium, as was described for the expression of galactosidase genes in *Aspergillus niger* [19]. The absence of pectin in compost would therefore abolish the induction of pectinase activity. The absence of CMC activity could be explained by the suggested link between production of cellulases by *A*. *bisporus* and fruiting body development [20], which is not yet initiated in PIII-16.

### Detailed analysis of enzymes from various compost extracts

For the second, more extensive screening, only CMC and xylan were selected as substrates based on the carbohydrate composition of compost previously described [1], which showed that cellulose and xylan are the two most abundant polysaccharides present in the compost used in this study. Moreover, transcriptomic study indicated up regulation of cellulases and xylanases in the compost during fruiting body formation in the compost [9]. In addition to wheat arabinoxylan (WAX), also birchwood xylan, substituted with glucuronic acid, was used as a substrate. Further, since high activity was detected towards mannan, galactomannan was added as a substrate, even though it is not a main component of compost. Screening of enzyme activities was assayed on PNP-substrates for exo-activities, as well as by PAHBAH for overall hydrolytic activity on the selected polysaccharides (see Materials and Methods). The results are discussed below for xylan, cellulose and mannan degrading enzyme activities.

The protein content of the extracts used was determined to be for PIIend, PIII-16, Filling, Pinning, 1^st^ flush and 2^nd^ flush, 6.2, 5.9, 7.1, 7.6, 9.4 and 7.8 mg protein g^-1^ dry matter of compost, respectively, indicating the highest protein content in the enzyme extract from 1^st^ flush. The pH of the extracts was measured as slightly acidic being 6.5, 6.2, 6.1, 6.3, 5.7 and 6.7, for PIIend, PIII-16, Filling, Pinning, 1^st^ flush and 2^nd^ flush, respectively. Further, conductivity of the extracts was measured and was found to be in a range between 3.3 and 4.7 mS cm^-1^. In addition, the protein profiles analyzed by SDS-PAGE gel electrophoresis were similar up to Pinning and showed very intense protein bands around 75 kDa. In contrast, the extract after 1^st^ flush, did not show the intense band around 75 kDa, but multiple less intense bands in the range of 50–75 kDa (S1 Fig).

#### Xylan degrading activities

**Fig 2:**
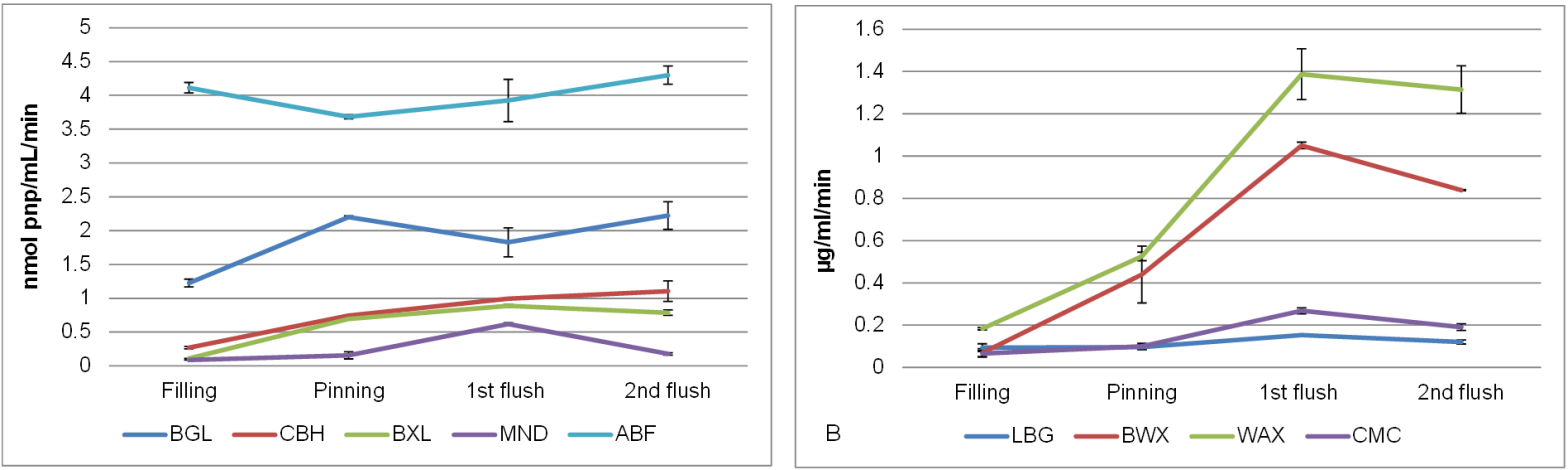
Relative enzyme activities in water extracts (4 h incubation) of various stages of mushroom production. A: exo-activities (in nmol PNP per ml per min). B: total hydrolysis based on reducing sugar determination (in **μ**g ml^-1^). BGL = β-glucosidase, CBH = cellobiohydrolase, BXL = β-xylosidase, ABF = α-arabinofuranosidase, MND = β-mannosidase. BWX = birchwood xylan, WAX = wheat arabinoxylan, CMC = carboxymethyl cellulose, LBG = locus bean gum.

The overall hydrolytic enzyme activity tested after incubation for 4h showed similar trends for WAX and birchwood xylan (Fig 2). Enzyme activity on both substrates was lowest in Filling and increased over time with the highest activity in 1^st^ flush. After that the activity tended to decline for about 20% in 2^nd^ flush. The same trend was observed after incubation for 24 h for both substrates. After 24 h, the concentration of reducing ends was found to be up to 4 times higher compared to incubation of 4h indicating that for tested enzymatic activities there is no substrate depletion. Concentration of reducing ends released from birchwood xylan was between 51 and 547 μg Xyl ml^-1^, and from WAX between 142 and 833 μg Xyl ml^-1^ (from 2 mg ml^-1^ of substrate). Similar to the 4h incubation, the 1^st^ flush-extract showed the highest activity on the two xylans tested; approximately 13% (birchwood xylan) and 17% (WAX) of the reducing ends were released from the substrate. This xylan degrading activity correlated well with the lower xylan content analyzed in the compost (Table 2), and indicated the presence of good hydrolytic activities towards xylan in the compost during fruiting body formation. The activity of β-xylosidase and α-arabinofuranosidase was also assayed on PNP-monosaccharides after a 4 h incubation (Fig 2). The β-xylosidase activity was lowest (0.1 nmol pnp ml^-1^ min^-1^) in the extract of Filling and it increased during fruiting body formation having the highest activity in compost collected after the first flush of mushrooms (1^st^ flush, 0.9 nmol pnp ml^-1^ min^-1^). During the growth of *A*. *bisporus* in compost (from filling until the stage when the entire second flush was harvested) the α-arabinofuranosidase activity was at least 4 times higher and more constant than the β-xylosidase activity (4.1 to 4.3 nmol pnp ml^-1^ min^-1^). Results of both overall and exo-enzyme activity demonstrated that the xylan degrading machinery of *A*. *bisporus* was active in the compost throughout the cultivation. The presence of these enzymatic activities is in line with the transcriptomic data obtained previously [9].

**Table 2:** Total carbohydrate content, carbohydrate molar composition and xylan degree of substitution of compost samples obtained at PII, PIII-16, after filling, after pinning, after 1^st^ and after 2^nd^ flush.

	PII^a^	PIII-16^a^	Filling^b^	Pinning^b^	1^st^flush^b^	2^nd^flush^b^
Total carbohydrates (% w/w)^c^	26±1	27±1	22±1	23±1	18±1	16±1
Carbohydrate composition (molar %)^d^						
Arabinose	5	5	6	4	6	6
Xylose	34	35	34	27	25	24
Mannose	1	3	5	6	9	8
Galactose	2	2	3	2	3	3
Rhamnose	2	2	1	1	2	1
Glucose	52	50	44	53	47	49
Uronic acid	4	5	6	6	8	8
Degree of substitution						
Ara/Xyl^e^	15	14	17	16	23	26
GlcA/Xyl^e^	11	13	18	21	31	35

^a^PII: Phase II compost; PIII-16 is Phase III compost after 16 days of mycelium growth (adapted from Jurak et al. [1]).

^b^Filling: compost after filling of compost beds at the farm, Pinning: after mushroom pins appear, 1^st^ flush: after first flush of mushroom was collected, 2^nd^ flush: spent compost, after 2^nd^ flush of mushrooms was collected.

^c^Weight percentage is based on dry matter of composting phases.

^d^As anhydro-sugars; STDEV < 0.5 for all samples.

^e^Ratio mol substituents/100mol of xylosyl residues; abbreviations: Ara, arabinosyl; Xyl, xylosyl; GlcA, glucuronic acid.

**Fig 3:**
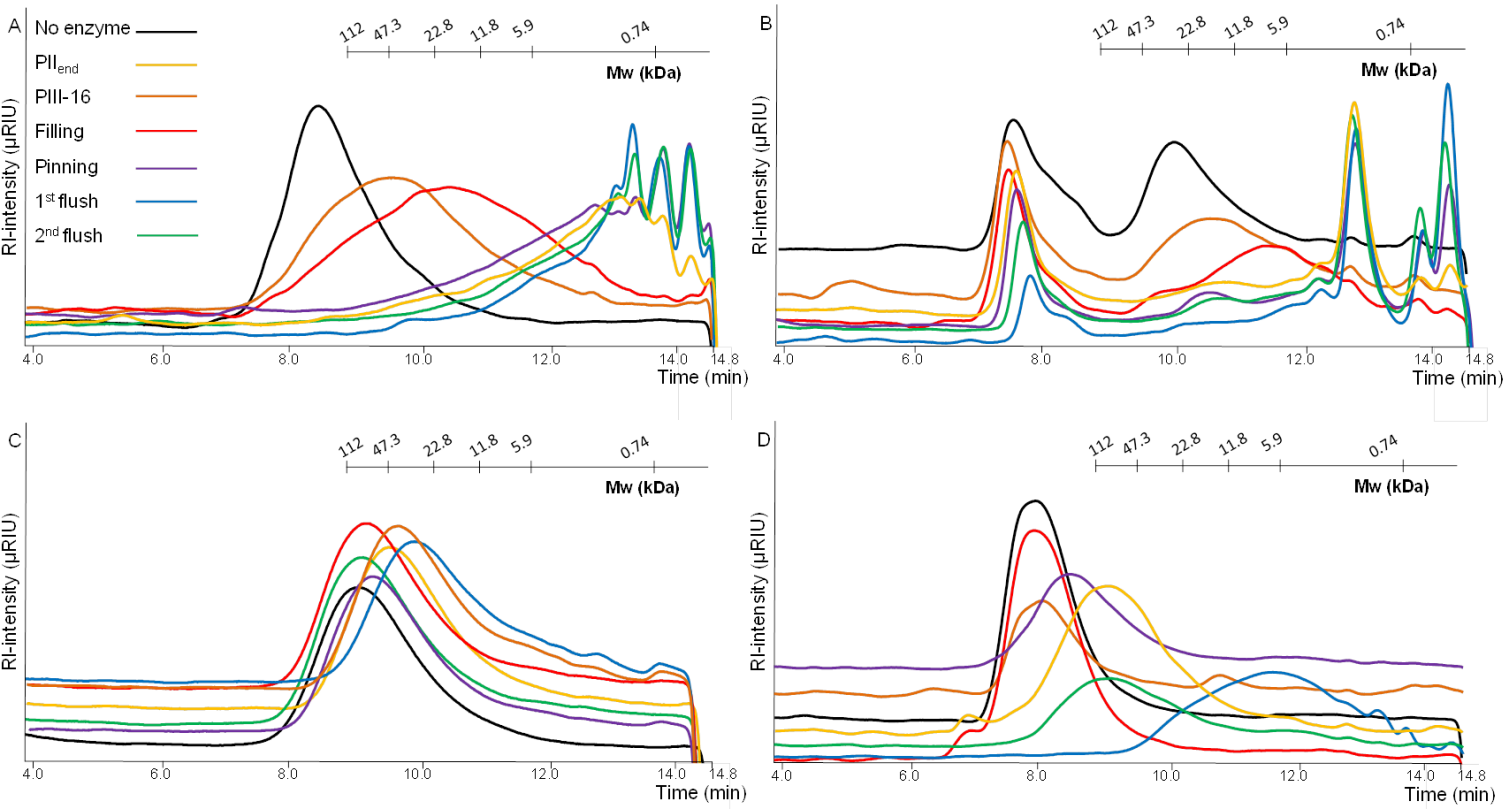
High performance size exclusion profiles of wheat arabinoxylan (A), birchwood xylan (B), carboxymethyl cellulose (C) and galactomannan (D), after degradation with enzyme extracts obtained from compost of different stages of mushroom growth (24 h incubation).

**Fig 4:**
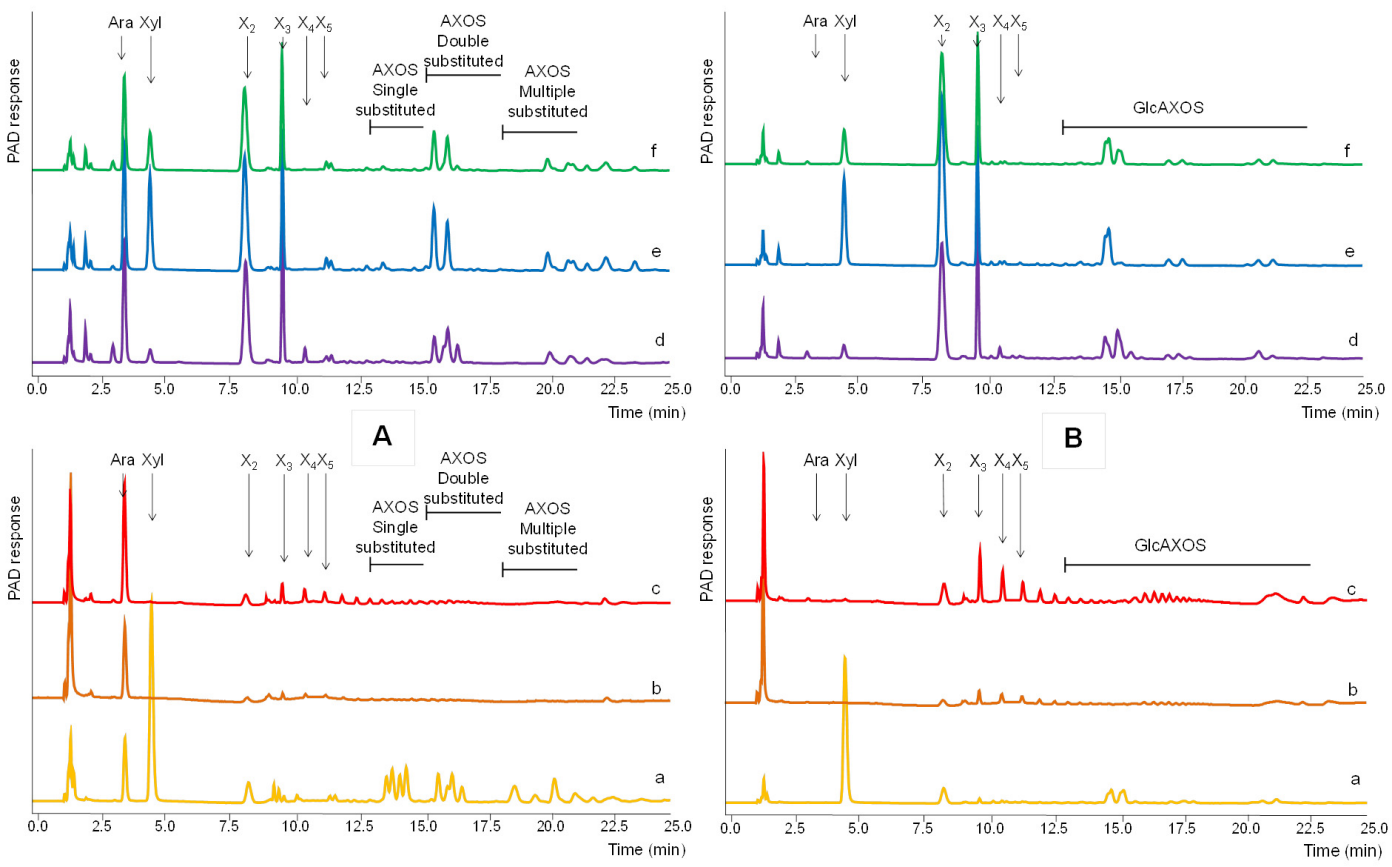
HPAEC elution profile of WAX (A) and birchwood xylan (B) incubated (24h) with a: PII_end_, b: PIII-16, c: Filling, d: Pinning, e: 1^st^ flush and f: 2^nd^ flush extracellular enzymes. Ara = arabinosyl, Xyl = xylosyl, X_2_ = xylobiose, X_3_ = xylotriose, X_4_ = xylotetraose, X_5_ = xylopentaose, AXOS = oligomers substituted with arabinose. GlcAXOS = oligomers substituted with 4-*O*-methyl-glucuronic acid.

Analysis of the xylans, before and after hydrolysis with the enzyme extracts, with HPSEC and HPAEC gave more detailed information about the mode-of-action of the various enzyme extracts used (Figs 3 and 4). The enzyme extract obtained from compost without mycelium (PIIend) completely degraded polymeric WAX into monomeric and small oligomeric compounds with a molecular mass <12 kDa (Fig 3). Over the period of mushroom growth (PIII-16 to 2^nd^ flush) two trends could be observed: partial and complete degradation of WAX. Partial degradation was obtained with extracts from PIII-16 and Filling, and the relatively large amounts of high molecular weight xylan that remained after the incubation indicated limited activity of endo-xylanases. This could be due to a change in the source of hydrolytic enzymes, from bacterial origin (PIIend) to *A*. *bisporus* (PIII-16 and Filling). Complete degradation of high molecular weight WAX was obtained with enzyme extracts from the period of fruiting body formation (Pinning to 2^nd^ flush) indicating an increase in the activity of xylanases. Again, maximum activity was achieved by the 1^st^ flush-extract. These results were in line with the results obtained by the reducing end assay (PAHBAH) after 24 h.

Degradation of 4-*O*-methyl-glucuronic acid substituted xylan (birchwood xylan, Fig 3) followed the same trend as was observed for WAX hydrolysis, although less pronounced. However, for birchwood xylan, high molecular weight material was still present after hydrolysis with the 1^st^ flush-extract, suggesting that the extracted enzymes were not able to completely degrade xylan substituted with 4-*O*-methyl-glucuronic acid.

The HPAEC profiles of WAX degraded by extracellular enzymes from the compost (24 h) are presented in Fig 4. WAX hydrolyzed with the PIIend-extract (Fig 4) resulted in relatively high amounts of xylose, indicating β-xylosidase next to endo-xylanase activity. Nevertheless, some small amounts of xylobiose and xylotriose remained. Further, relatively low amounts of arabinose were released (S1 Table). Together with the presence of xylo-oligomers substituted with arabinose this indicated a poor arabinofuranosidase activity in PIIend. The HPAEC chromatogram of WAX incubated with extracts from PIII-16 and Filling (Fig 4) showed only low amounts of arabinose, no xylose and very small amounts of xylan-oligomers. This was also in line with the results obtained by HPSEC (Fig 3), which showed mainly large molecular weight xylan remaining.

These results matched with previous research [1] suggesting that mainly partial degradation of xylan occurs during 16 days of mycelium growth. This increases the solubility of xylan and results in a relatively easy to degrade carbohydrate source available during fruiting body formation. Indeed, WAX degraded with extracts from Pinning, 1^st^ flush and 2^nd^ flush showed much more xylose and arabinose as well as xylobiose and xylotriose than from PIIend, PIII-16 and Filling (Fig 4), being the result from a nearly complete WAX hydrolysis as was also detected on HPSEC (Fig 3).

Although during the period of fruiting body formation an increase in β-xylosidase activity is observed, relatively high amounts of xylo-oligomers remained. However, no single substituted xylo-oligomers were detected. This suggested the presence of an efficient α-arabinofuranosidase in the compost in these stages that is able to release arabinosyl units present as single substituents on the xylan backbone. This xylanase and arabinofuranosidase activity correlates well with the upregulated xylanase- and arabinofuranosidase-encoding genes at the stage of the first flush of *A*. *bisporus*, as published previously [9].

**Fig 5:**
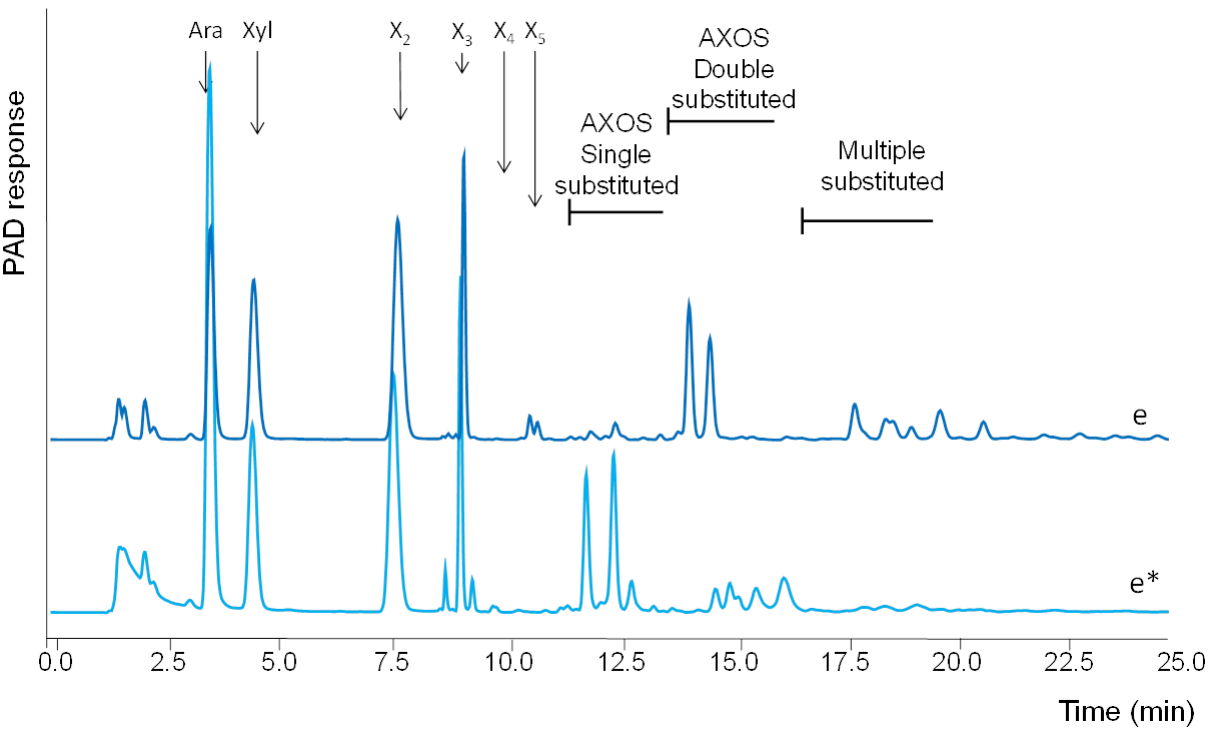
HPAEC elution profile of WAX incubated (24h) first with 1^st^ flush (e) and then sequentially with pure GH43 AXH-d3 arabinofuranosidase (e*) [17]. Ara = arabinosyl, Xyl = xylosyl, X_2_ = xylobiose, X_3_ = xylotriose, X_4_ = xylotetraose, X_5_ = xylopentaose, AXOS = oligomers substituted with arabinose.

In contrast, double substituted xylo-oligomers remained (Fig 5). Apparently, arabinofuranosidase activity able to release arabinosyl units from doubly substituted xylooligomers was lacking. Two of such specific arabinofuranosidases have been characterized, both belonging to the GH43 family (CAZy, www.cazy.org), one produced by *Humicola insolens* [21] and one by *Bifidobacterium adolescentus* [22]. For *A*. *bisporus* [9] putative genes encoding enzymes belonging to the family GH43 were found to be up regulated in compost (1^st^ flush) compared to plate grown mycelium, but, it is shown in a phylogeny tree that these GH43 genes are not likely to be active towards the doubly substituted xylan parts [23]. Therefore, it could be speculated that adding these specific enzymatic activities could lead to better de-branching of xylan and improve overall xylan degradation during the growth of *A*. *bisporus* in compost in time.

Fig 4 shows the hydrolysis products of birchwood xylan substituted with 4-*O*-methyl-glucuronic acid analyzed with HPAEC. No free 4-*O*-methyl-glucuronic acid was detected in any of the digests. Apparently, none of the enzyme extracts was able to release 4-*O*-methyl-glucuronic acid from xylan or xylan oligomers indicating that the α-glucuronidase activity was either not present or excreted in non-detectable amounts. Nevertheless, *A*. *bisporus* contains two genes predicted to encode α-glucuronidases activity, but these were both not significantly expressed during the growth of A. bisporus in compost as analyzed in the same batch of samples as used in this research [23]. Also, α-glucuronidase activity was previously detected after growing *A*. *bisporus* mycelium on beechwood xylan. This suggests that the commercial growth conditions for *A*. *bisporus* result in a different physiology than was previously described for this species on beechwood xylan [24].

In conclusion, substantial xylanase activity was observed (based on degradation of WAX observed on HPSEC chromatograms (Fig 3) in enzyme extracts obtained from various growth stages during the commercial cultivation of *A*. *bisporus*. For complete saccharification of xylan, more efficient β-xylosidase activity, α-glucuronidase and arabinofuranosidase activity able to release arabinosyl units from doubly substituted xylooligomers activity is needed than was produced in these samples. These findings were found to correspond to the accumulation of xylan residues substituted with glucuronic acid and two arabinosyl- residues in the compost during fruiting body formation of *A*. *bisporus* (Jurak et al., 2015 [unpublished data]). This accumulation of glucuronic acid and arabinosyl- residues was found in water- insoluble part of compost indicating that even though non-extracted enzymes were not studied in this research, insoluble structures remaining support the conclusion that these specific enzymatic activities are not present in the compost, as was found in the water soluble enzyme extracts. Based on these findings it is proposed that addition of these enzymatic activities, e.g. at Filling, could improve xylan degradation in the compost. However, whether this would lead to higher mushroom yields, remains unclear.

#### Cellulose degrading activities

Overall, cellulase activity tested on CMC after 4 h confirmed the activity of cellulases in the compost throughout the fruiting body formation as previously reported by Wood and Goodenough [25] (Fig 2). About 16 μg Glc ml^-1^ reducing ends were released from CMC with the Filling-extract and the activity increased slightly in compost obtained after mushroom pins appeared (Pinning, 23 μg Glc ml^-1^). Highest cellulase activity was observed in the compost-extract obtained after the first flush of mushrooms (1^st^ flush, 64μg Glc ml^-1^) and the activity decreased slightly after the second flush was collected (2^nd^ flush, 46 μg Glc ml^-1^).

Cellobiohydrolase activity (4h incubation) of was lowest in the extract from Filling (0.3 nmol pnp ml^-1^ min^-1^) and increased throughout the fruiting body formation having the highest activity (1.1 nmol pnp ml^-1^ min^-1^) in 2^nd^ flush (Fig 2). In the extract from Filling, β-glucosidase activity was the lowest (1.2 nmol pnp ml^-1^ min^-1^), while after pinning (Pinning), activity was much higher (2.2 nmol pnp ml^-1^ min^-1^) and it decreased again in 1^st^ flush (1.8 nmol pnp ml^-1^ min^-1^). In the 2^nd^ flush extract, β-glucosidase activity was the same as after pinning.

HPSEC-chromatograms (24 h incubation) showed that all compost extracts were able to partially degrade CMC. In comparison with xylan hydrolysis CMC hydrolysis was much lower, as was observed by the rather large amount of high molecular weight (Mw) material remaining after the incubation. This could be due to the difference in structure of CMC and the cellulose present in compost. After 16 days of mycelium growth (PIII-16) cellulase activity decreased compared to PIIend. From filling (Filling) to the first flush (1^st^ flush) cellulase activity increased and after the second flush (2^nd^ flush) a slight decrease in activity was detected (Fig 3).

Overall, the trend of CMC hydrolysis by enzyme extracts from the various growth stages observed with HPSEC was in line with the trend observed for the same samples analyzed by PAHBAH assay after 4 (see Fig 2) and 24 h (not shown). Previously, it was reported that the onset of fruiting body formation is accompanied by an increase in cellulase activity, in particular endo-glucanase activity [25]. Also, during fruiting body formation the rate of cellulose and hemicellulose degradation was analyzed to be higher compared to mycelium grown compost [26]. The results of our study confirm this, since we obtained high cellulase and hemicellulase activities during pinning stage, as well as when the entire first and second flushes were harvested. Our data also fit with the previous transcriptomics study of *A*. *bisporus*, which demonstrated that cellulase encoding genes were upregulated in the compost compared to plate grown mycelium [9]. Xylanase activities were higher than most cellulase activities in compost, which confirms a previous study [5], and suggested that xylan is an important growth substrate for *A*. *bisporus*, especially during fruiting body formation. As xylan consists mainly of the pentoses xylose and arabinose, this correlates well with the upregulation of most of the pentose catabolic pathway genes, required for conversion of these pentoses, in mycelium grown compost compared to plate grown mycelium [9].

#### Mannan degrading activities

Fig 3 shows HPSEC profiles of galactomannan degraded by enzyme extracts (24 h incubation). For both the exo-activity and the overall enzyme activity (Fig 2) the same trend was observed. In extracts from Filling and Pinning very low mannanase activity was detected. Nevertheless, in 1^st^ flush a remarkable increase in both endo- and exo-activity was observed. In 2^nd^ flush the overall mannanase activity decreased (Fig 3) and exo-activity decreased for 70% (Fig 2). Overall, this trend was similar to the one observed for cellulases and xylanases.

### Compost composition and carbohydrate structures

Table 2 shows that for all samples the main polysaccharides present consisted of xylosyl (34–24 mol%) and glucosyl (52–49 mol%) residues, as was previously described for wheat straw based compost [1,2].

PII compost contained about 26% (w/w DM) of carbohydrates and during the 16 days of mycelium growth, the total carbohydrate content, including both plant and fungal biomass carbohydrates, remained the same [1]. This indicated that only low amounts of carbohydrates were metabolized from the compost carbohydrates by the microbial population in PIII-16, which was also concluded from the rather low enzyme activities present in this phase, being mainly endo-activity. Further, as previously mentioned, the partial degradation of compost carbohydrates in PIII-16, delivered soluble carbohydrates for *A*. *bisporus* needed during fruiting. For the growth of *A*. *bisporus*, complete saccharification of compost carbohydrates during early stages of mycelium growth is not favored, because increasing levels of monosaccharides will promote microbial growth over subsequent *A*. *bisporus* growth.

After compost is spent (2^nd^ flush) total carbohydrate content decreased to about 16%. Previously, it was reported that during mycelium growth and fruiting of A. *bisporus* carbohydrate content decreases to 11% [2]. This difference may be, among other, due to variations in the origin of raw materials (Australia versus Europe) and the composting process (e.g. mycelium was grown for 4 weeks versus 16 days, before addition of casing layer and compost was considered spent after 1^st^ flush of mushrooms was collected) [2]. Comparing the molar carbohydrate composition of the samples obtained during pinning up to spent compost (2^nd^ flush), a gradual decrease in xylosyl and an increase in arabinosyl and uronic acids, most likely glucuronic acid residues, was observed (Table 2). The decrease in xylosyl residues correlated with the maximum activity of xylan degrading enzymes observed. Overall, the amount of total xylan substituents in the compost increased two times from Pinning to 2^nd^ flush (Table 2). This suggests the inability of *A*. *bisporus* to degrade xylan substituted with glucuronic acid or with two arabinosyl residues per xylosyl residues, leading to an accumulation of these recalcitrant xylan structures. Therefore, it can be speculated that in spent compost (2^nd^ flush) mainly xylan substituted with glucuronic acid or two arabinosyl residues per xylosyl residue is present.

## Conclusions

In all enzyme extracts from compost during growth of *A*. *bisporus* the activity of endo-xylanase and β-xylosidase activities was present and to a lesser extent of glucanase. Maximal overall enzymatic activity was observed after the first flush of mushrooms. In contrast, α-glucuronidase activity and arabinofuranosidase activity able to remove arabinosyl residues from doubly substituted xylose residues was absent in these extracts. As a result, the degree of substitution of xylan with both arabinosyl and glucuronic acid significantly increased during fruiting body formation of *A*. *bisporus*. Exploring the options to apply these missing xylan de-branching enzymes directly to the compost or developing *A*. *bisporus* strains that include these activities in their enzyme-machinery may improve commercial mushroom production.

## Supporting Information

S1 Fig Protein profiles on SDS PAGE. M: marker, Extracts of 1: PIII-16, 2: Filling, 3: Pinning, 4: 1^st^ flush, 5: 2^nd^ flush.
(TIF)

S1 Table Released mono and oligosaccharides from wheat arabinoxylan and birchwood xylan after 24 h digestion with extracellular enzymes from different compost phases, analyzed by HPAEC. (DOCX)
