# Supplementary figures and images for: Compost Grown Agaricus bisporus Lacks the Ability to Degrade and Consume Highly Substituted Xylan Fragments
Source: PLoS One. 2015 Aug 3;10(8):e0134169. doi: 10.1371/journal.pone.0134169 (PMC4523207; doi:10.1371/journal.pone.0134169)

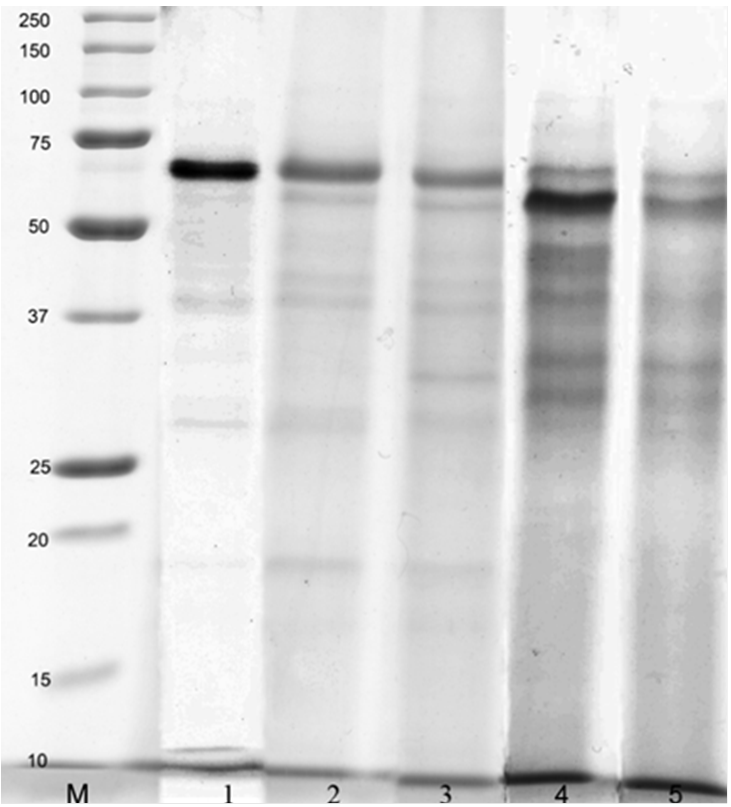

Supplement: S1 Fig — M: marker, Extracts of 1: PIII-16, 2: Filling, 3: Pinning, 4: 1st flush, 5: 2nd flush. (TIF) [file pone.0134169.s001.tif]
