# Supplementary material for: Compost Grown Agaricus bisporus Lacks the Ability to Degrade and Consume Highly Substituted Xylan Fragments
Source: PLoS One. 2015 Aug 3;10(8):e0134169. doi: 10.1371/journal.pone.0134169 (PMC4523207; doi:10.1371/journal.pone.0134169)
Supplement: S1 Table — (DOCX) [file pone.0134169.s002.docx]

Supplementary table 1. Released mono and oligosaccharides from wheat arabinoxylan and birchwood xylan after 24h digestion with extracellular enzymes from different compost phases, analyzed by HPAEC.

| Concentration (µg released/mg of substrate) | | | | | | | |
| --- | --- | --- | --- | --- | --- | --- | --- |
|  | Xyl | Ara | X_2_ | X_3_ | X_4_ | GlcA | Total |
| **WAX** |  |  |  |  |  |  |  |
| PII_end_ | 102 | 23 | 19 | 0 | 0 | n.a | 144 |
| PIII-16 | 0 | 12 | 2 | 0 | 0 | n.a | 14 |
| Filling | 0 | 30 | 7 | 7 | 0 | n.a | 44 |
| Pinning | 8 | 57 | 124 | 106 | 12 | n.a | 307 |
| 1^st^ flush | 61 | 60 | 141 | 92 | 0 | n.a | 354 |
| 2^nd^ flush | 33 | 58 | 131 | 107 | 0 | n.a | 328 |
|  |  |  |  |  |  |  |  |
| **Birchwood xylan** | |  |  |  |  |  |  |
| PII_end_ | 202 | n.a | 36 | 6 | 0 | n.f. | 245 |
| PIII-16 | 0 | n.a | 4 | 5 | 0 | n.f. | 8 |
| Filling | 1 | n.a | 12 | 16 | 17 | n.f. | 47 |
| Pinning | 9 | n.a | 153 | 100 | 11 | n.f. | 273 |
| 1^st^ flush | 69 | n.a | 255 | 108 | 0 | n.f. | 432 |
| 2^nd^ flush | 30 | n.a | 187 | 117 | 0 | n.f. | 335 |

Xyl: xylose, Ara: arabinose, GlcA: glucuronic acid, X_2_: xylobiose, X_3_: xylotriose, X_4_: xylotetraose.

n.f.: not found, n.a.: not applicable.
